# Examining the asymmetric effects of internationalization factors on student satisfaction

**DOI:** 10.1038/s41598-025-17460-w

**Published:** 2025-08-29

**Authors:** Fan Liu, Xian-Jun Li

**Affiliations:** School of Education, Central China Normal University, No. 152, Luo Yu Road, Hongshan, Wuhan, 430079 Hubei People’s Republic of China

**Keywords:** Sino-foreign cooperative universities, Education internationalization, Topic modeling, Student satisfaction, Higher education, Psychology and behaviour, Information technology

## Abstract

Following the COVID-19 pandemic, an increasing number of Chinese students are choosing to pursue internationalized higher education domestically rather than studying abroad. However, current internationalization strategies are often not fully aligned with students’ evolving needs. This study examines the determinants of student satisfaction with internationalization practices at Sino-foreign Cooperative Universities (SFCUs), emphasizing both globally recognized and context-specific elements. The findings suggest that several formal aspects of internationalization, such as curriculum internationalization, institutional reputation, and mobility programs, as the most significant predictors of student satisfaction. Among these, a globally oriented curriculum stands out as the strongest determinant, emphasizing the importance of academic quality, cross-cultural relevance, and alignment with global competencies in influencing the student experience. Conversely, we found that more subjective aspects of internationalization, such as cultural integration and student management practices, had a less direct impact on satisfaction. Nevertheless, this study confirms the importance of the perceptions of fairness in resource allocation and student treatment, suggesting that equity and transparency in institutional practices are essential for fostering a sense of belonging. While formal language support did not emerge as a major predictor of satisfaction, this finding suggests that SFCUs should instead focus on fostering an immersive English environment, especially given students’ already high language proficiency. These findings provide valuable insights into how SFCUs can refine their internationalization strategies, emphasizing the need for an approach that balances global educational standards with the specific institutional contexts of the host country.

## Introduction

The significance of internationalization in higher education is widely recognized. Although no single definition is universally accepted, Knight’s^1^ conceptualization is frequently cited. Knight^1^ defines internationalization as the process of incorporating international, intercultural, and global dimensions into the goals, functions, and delivery of higher education. In recent decades, this concept has grown in prominence due to an increasingly interconnected world. Global competencies and cross-cultural understanding have become essential. As a result, universities worldwide have prioritized internationalization as a strategic objective. This growing emphasis reflects the recognition of several key benefits. One of the most widely acknowledged advantages of internationalization is its role in preparing graduates to develop cultural competence, thus equipping them with the necessary skills to navigate and succeed in diverse and globalized environments^2–4^. Furthermore, from an academic perspective, internationalization fosters research that tackles some of the world’s most pressing global challenges, including climate change, public health crises, and social inequality^5–7^. Beyond this, it enhances cultural diversity within academic communities, promoting inclusivity and broadening intellectual horizons^8–10^.

However, despite these recognized benefits, internationalization remains a complex process fraught with challenges. One of the most significant issues is the operation of internationalization within existing global power imbalances. Western paradigms frequently dominate the global academic sector, often marginalizing non-Western perspectives and imposing frameworks that do not adequately address the diversity of local contexts^6,11^. This imbalance raises important questions about the universality of internationalization practices and whether they truly meet the needs of non-Western societies. In educational psychology, the concept of cultural self-efficacy is crucial in understanding how students from different cultural backgrounds engage with foreign educational practices^12^. When the dominant educational models are rooted in Western traditions, students from non-Western backgrounds may experience a sense of alienation or lack of confidence in navigating systems that do not reflect their cultural values or learning styles^13^. Moreover, accessibility and equity in international education continue to be major concerns, as financial and logistical barriers disproportionately hinder participation from students in lower-income countries^14,15^. These challenges exacerbate existing inequalities and necessitate a more equitable approach to internationalization, one that addresses the structural constraints faced by underrepresented groups.

In China’s higher education sector, the development of internationalization policies is often driven by national-level priorities, faculty interests, or institutional goals^16,17^. However, these policies frequently fail to adequately consider the perspectives and experiences of students^18^, leading to a disconnect between top-down policy frameworks and the lived realities of those directly affected by internationalization strategies. Zapp and Lerch^19^ contend that higher education increasingly aligns with global cultural models, serving both national and broader global interests. This shift brings attention to a more inclusive approach that considers the diverse needs of students in the process of internationalization. By adopting a more student-centered perspective, universities can create an internationalization process that is not only academically rigorous but also socially responsible and culturally sensitive.

One significant challenge lies in the inadequate adaptation of internationalization strategies to the local context^20^, which can create a disconnect between policy intentions and student experiences. Transnational education in China has shown marked improvement in quality over time; however, earlier criticisms highlighted the limited educational resources and inconsistent standards in international partnerships^21^. Later evaluations, such as Li and Eyong^22^, acknowledge progress in addressing these issues, yet persistent challenges remain. A notable issue is the one-directional reliance on Western academic systems, which continues to dominate China’s internationalization practices^23^. This reliance often limits the incorporation of local cultural and intellectual traditions into international education models, potentially alienating students who seek a balance between global and local perspectives. As a result, some scholars advocate for a more balanced approach to internationalization—one that incorporates Chinese philosophical and cultural foundations alongside global best practices^17,24^.

Given these complexities, developing context-sensitive internationalization strategies specifically tailored to the local environment is crucial. This study focuses on Sino-foreign cooperative universities (SFCUs), which play a pivotal role in China’s higher education internationalization agenda^25^. SFCUs, as key institutions driving these efforts^26,27^, are at the forefront of implementing internationalization initiatives. Therefore, it is essential to understand and address the unique needs of their students. However, the effectiveness of current strategies often comes into question, particularly regarding student satisfaction and their perceived value of the internationalized education they receive. This raises concerns about whether these policies are genuinely effective in creating meaningful educational experiences or merely serve to fulfill administrative and institutional objectives. A significant gap in the existing literature on internationalization in Chinese higher education lies in its predominant focus on qualitative methodologies (e.g.^21,23,28^). While these studies provide valuable descriptive insights into the processes, challenges, and outcomes of internationalization, they often fail to deliver robust quantitative assessments. This absence of empirical measurement makes it difficult to evaluate the relative importance and impact of different internationalization strategies, especially from the perspective of students. This study attempts to bridge this gap by addressing the following research question: What is the impact of common internationalization approaches and context-specific strategies on student satisfaction with internationalization practices in SFCUs?

This study seeks to explore the impact of both widely adopted internationalization approaches and often overlooked contextual factors on student satisfaction with internationalization practices in SFCUs. In addition, this study incorporates advanced text mining techniques to discover insights that may not be captured through closed-ended questions alone. By doing so, this research makes several significant theoretical and practical contributions to the field of internationalization in higher education, particularly within the context of SFCUs. Theoretically, the study advances the existing literature by exploring the impacts of internationalization practices on student satisfaction. It achieves this by developing and validating a structural equation model (SEM) to quantify the relationships between internationalization strategies and student satisfaction, while also integrating context-specific variables, such as cultural factors and student management styles. Furthermore, the use of the Latent Dirichlet Allocation (LDA) text mining technique to analyze student feedback introduces a novel methodological approach to educational research, bridging qualitative and quantitative insights. Practically, the research offers actionable recommendations for policymakers and educators. It provides a roadmap for prioritizing and refining internationalization strategies to better align with student needs and expectations. Importantly, the findings emphasize the necessity of tailoring global practices to the unique local context of Chinese universities, striking a balance between cultural integration and educational values. By addressing these gaps, the study contributes to the development of more effective and inclusive internationalization policies in SFCUs, ensuring that these institutions can deliver meaningful and context-sensitive educational experiences.

## Theoretical background and hypothesis

### Theoretical foundation

This study is grounded in two conceptual frameworks that facilitate the exploration of the internationalization process in higher education. The first framework is derived from Knight’s^29^ seminal definition of internationalization, which is widely acknowledged as one of the most influential and comprehensive models in the educational field. Knight’s framework serves as a foundational guide for understanding how higher education institutions operationalize internationalization across various dimensions, including curriculum development, student exchange programs, and institutional policies. However, it is important to point out that the internationalization process is not solely determined by overarching frameworks; it is significantly influenced by local contexts and institution-specific factors. These include aspects such as organizational structure, strategic objectives, and culture^30,31^. Increasingly, scholars emphasize that the interpretation and implementation of internationalization are profoundly sensitive to contextual variables^9,32^, resulting in variability across different institutions, nations, and cultural settings. To address this complexity, this study seeks to extend Knight’s framework by integrating additional contextual factors that affect the internationalization process. Specifically, we propose the inclusion of three critical dimensions—Student Management Styles, Perceived Equality, and Cultural Integration. Existing research highlights the distinctive characteristics of Chinese higher education, which inform these dimensions.

### Hypothesis development

#### Curriculum internationalization

Curriculum development is a cornerstone in the pursuit of internationalization in higher education. Cole^33^ introduces the concept of “logics of academic consumerism,” where faculty members are viewed as producers, students as consumers, and curricula as commodities that evolve in response to students’ changing interests. From the students’ perspective, an internationalized curriculum equips them with the skills and perspectives essential for success in a globalized world. It fosters cultural understanding^34^, enhances critical thinking^35^, and prepares students to engage with global issues effectively^36^. Specifically, Fragouli^36^ emphasizes the necessity of internationalizing the business management curriculum to better prepare students for the challenges and opportunities presented by a global marketplace. Similarly, Leask^37^ asserts that an internationalized curriculum aligns broader institutional objectives with student learning, thereby making education more relevant and inclusive. However, this process often requires rethinking traditional Western-centric educational approaches^38^. Curriculum internationalization involves several key aspects, including the integration of global and intercultural perspectives^39^, thoughtful curriculum design and content integration^40^, and transformative learning experiences^41^. Considering the role of curriculum internationalization, including the direct benefits to students, we propose the hypothesis:

##### H1

*Curriculum internationalization is positively related to students’ satisfaction with internationalization in SFCUs.*

#### Language support

Although language support is often regarded as an integral part of the curriculum, in this study, it primarily refers to the delivery of knowledge within a specific field of study. Language support, on the other hand, includes both in-class and out-of-class assistance, serving as a crucial measure to help students succeed academically. This support is essential in SFCUs, which not only aids in overcoming immediate academic challenges but also fosters multilingualism, a key component of global competence^42^. Providing adequate language support is crucial in SFCUs, where many courses often adopt English as the medium of instruction (EMI). Galloway and Ruegg^43^ highlight that comprehensive language and academic skills support are crucial for the success of EMI programs, as they address the dual needs of content mastery and language proficiency. Given that many Chinese students frequently lack the necessary preparation to participate effectively in English-taught courses^44^, this is especially significant. Given the importance of language support in facilitating internationalization, particularly in contexts where English is not the native language, we propose the following hypothesis:

##### H2

*Language support is positively related to students’ satisfaction with internationalization in SFCUs.*

#### Student mobility programs

Student mobility programs are a key component of the internationalization of higher education and have a significant impact on students’ satisfaction with these efforts. These programs, which include study abroad opportunities, exchange programs, and internships in foreign countries, offer students the chance to immerse themselves in different cultural and educational environments^45^. This immersion is necessary to cultivate intercultural competence and global awareness, which are vital skills in a globalized world^46^. Research shows that participation in student mobility programs is positively associated with higher levels of student satisfaction with internationalization^47^. According to Knight^8^, students who engage in mobility programs often report enhanced personal and academic development, greater cross-cultural understanding, and a stronger sense of global citizenship. These outcomes contribute to a more fulfilling educational experience and align closely with the goals of internationalization. Moreover, students highly value the experiential learning opportunities provided by student mobility programs. This hands-on experience is often cited as a significant factor in students’ overall employability, particularly in internationalized programs^48^. In the context of SFCUs, where the aim is to blend the strengths of both domestic and international educational practices, student mobility programs are especially valuable. Based on this understanding, we propose the hypothesis:

##### H3

*Mobility programs are positively related to students’ satisfaction with internationalization in SFCUs.*

#### Global reputation of foreign universities

According to Hazelkorn^49^, students’ perceptions of a university’s global standing can impact their overall educational experience, including their sense of pride and confidence in their qualifications. In SFCUs, where students often seek to benefit from the prestige of a foreign education while studying domestically, the global reputation of the foreign partner can be an important factor in their satisfaction. In the context of internationalization, the global reputation of a foreign partner university can also influence the perceived legitimacy and credibility of the cooperative programs offered. Altbach and Knight^5^ argue that in cross-border education, the reputation of the foreign partner is a key determinant of the program’s attractiveness and the student’s overall experience. This is particularly true in SFCUs, where students expect the same level of academic rigor and global recognition as they would receive if they were studying abroad. Furthermore, the global reputation of the foreign partner university can enhance the perceived value of the degree awarded by the Sino-foreign cooperative institution. Students often consider the international recognition of their degree as a crucial aspect of their education, influencing their future career opportunities both domestically and internationally^26^. As noted by Marginson^50^, the global visibility and reputation of a university can significantly impact students’ satisfaction, particularly in institutions that are part of international collaborations. Given these factors, we propose the hypothesis:

##### H4

*Global reputation of foreign universities is positively related to students’ satisfaction with internationalization in SFCUs.*

#### Campus environment

The physical aspects of the campus environment play a crucial role in facilitating the internationalization process in the context of SFCUs. A well-designed campus that integrates international architectural styles, learning spaces, and facilities can create an inclusive and globally oriented atmosphere^30^. Studies have shown that physical spaces that support student engagement, such as open learning areas, multicultural centers, and modern technological infrastructure, contribute to enhanced student satisfaction and internationalization outcomes^51,52^. Moreover, the internationalized campus environment helps facilitate the use of English as the EMI, contributing to the rapid development of students’ language skills^53^. Therefore, we proposed the hypothesis:

##### H5

*Campus environment internationalization is positively related to students’ satisfaction with internationalization in SFCUs.*

#### Student management styles

The internationalization of student management styles in SFCUs should be considered a factor that may shape students’ perception of internationalization. Previous studies have indicated that internationalized management practices, such as flexible academic structures, cross-cultural interactions, and student-centered governance, contribute to higher levels of student satisfaction and engagement in cross-border educational settings^54,55^. This approach contrasts with more hierarchical and rigid local management styles traditionally found in Chinese institutions, which may limit student autonomy and flexibility^56^. However, international approaches such as involving students more in the decision-making process and offering diverse extracurricular activities are moving in a more international direction^57^. Therefore, we proposed the hypothesis:

##### H6

*The internationalization of student management styles in SFCUs positively influences student satisfaction with internationalization.*

#### Perceived equality

Ensuring equitable treatment for all students, regardless of their nationality, is key to creating a truly international and inclusive educational environment. When students perceive equality in institutional policies and practices, their overall satisfaction with the internationalization process increases, contributing to a positive educational experience. In many Chinese universities, foreign students are often afforded privileges such as superior accommodations and additional benefits^58^, which can create perceptions of inequality among the student body. This perceived inequity can undermine the sense of community and inclusivity that is fundamental to successful internationalization. As Marginson^59^ argues, fairness and equality are central to the legitimacy of educational institutions, and perceived inequities can lead to dissatisfaction and disengagement among students. Anderson^60^ emphasizes that equality in education involves providing all students with the resources and opportunities necessary to achieve their full potential. In SFCUs, this means creating an environment where both domestic and international students have equal access to high-quality educational experiences and support services. Additionally, transparent communication and consistent application of policies are key to maintaining perceptions of equality. Tannock^61^ notes that when institutions communicate their policies and ensure that they are applied consistently to all students, it enhances trust and satisfaction among the student body. Given these considerations, the perception of fairness is a crucial determinant of students’ satisfaction with internationalization in SFCUs. We propose the hypothesis:

##### H7

*Perceived equality is positively related to students’ satisfaction with internationalization in SFCUs.*

#### Cultural integration

Cultural integration plays a crucial role in balancing the predominantly Western-centric approach to internationalization in SFCUs. Previous studies have criticized the tendency to overly emphasize Western educational models and values, often sidelining the unique cultural and educational contexts of the host countries. For instance, Guo et al.^23^ argue that internationalization in Chinese universities has often been perceived as synonymous with Westernization, which risks undermining indigenous educational philosophies and practices. Cultural integration, which harmonizes local cultural values with global perspectives, provides a framework for achieving balanced internationalization^13^. By prioritizing this dual engagement, Chinese higher education institutions can enhance mutual understanding across cultures while mitigating the risk of becoming culturally adrift—a dilemma where institutions fail to embody either local identity fully or global relevance^62^. This approach not only addresses the tensions inherent in globalization but also positions cultural synergy as a strategic imperative for sustainable international engagement. As Zhang^27^ points out, integrating both Chinese and foreign educational characteristics in Sino-foreign cooperative programs enhances innovation in teaching and learning. Thus, we propose the following hypothesis:

##### H8

*Cultural integration is positively related to student satisfaction with internationalization in SFCUs.*

**Fig. 1:**
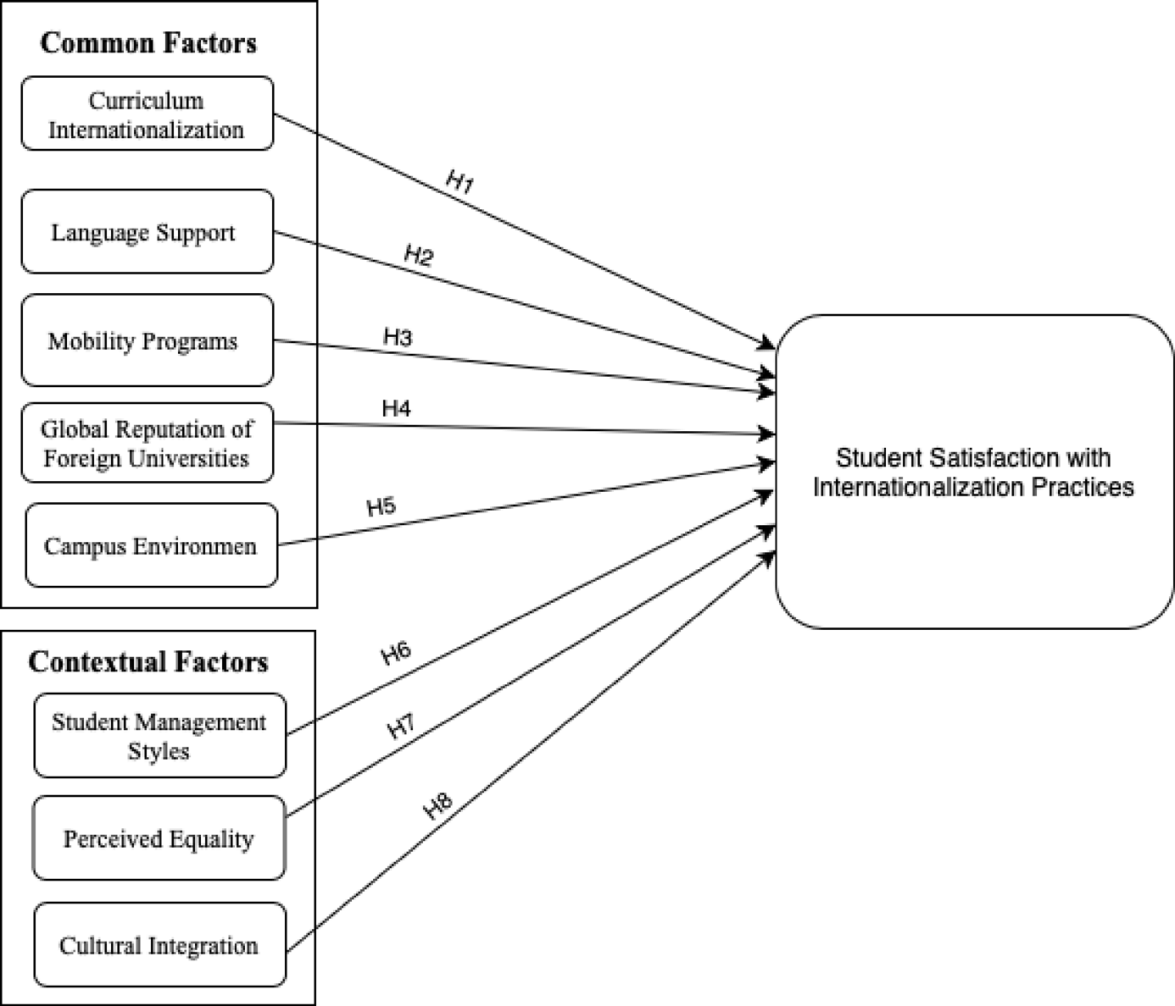
The research model.

The above hypotheses to examine the effect of common internationalization factors and contextual factors on students’ satisfaction with internationalization practices in SFCUs are shown in Fig. 1.

## Method

### Data collection

The data was collected from three SFCUs in Eastern China. Students were initially identified and contacted through popular social media platforms, such as Baidu Tieba, Rednote, Douyin, and Bilibili. Students widely use these platforms. To encourage participation, small rewards were offered. Students who shared or filled in the online questionnaire within their student communities received gift vouchers or the chance to win an electronic device. The online questionnaire consisted of two primary sections. The first section provided an overview of the research purpose, including the study’s objectives and its significance within the field of internationalization in higher education. This section also detailed the ethical guidelines governing the research, emphasizing the voluntary nature of participation, the guarantee of confidentiality regarding individual responses, and the participants’ right to withdraw from the study at any time without penalty. The second section of the questionnaire contained a series of detailed questions designed to address the research objectives. These questions collected a range of data points, including demographic information, behavioral patterns related to internationalization activities, and attitudinal data concerning various aspects of internationalization practices. To capture a more in-depth understanding of student perspectives, a mixed-methods approach was used, incorporating both quantitative items (e.g., Likert scales) and open-ended qualitative questions, with a minimum response length of 30 Chinese characters for the latter.

### Participants

All the participants are enrolled in full-time programs at SFCUs. The demographic details of the participants are presented in Table 1. Of the 531 students, 39.4% (n = 209) were male, and 60.6% (n = 322) were female. Most of them (78.3%, n = 416) were undergraduates, while 21.7% (n = 115) were postgraduate students. In terms of age distribution, the majority of students (65.6%, n = 348) were between the ages of 18 and 24, followed by 29.9% (n = 159) in the 25–30 age group, and 4.5% (n = 24) were aged 31 or older. 58.4% (n = 310) of the participants enrolled in liberal arts programs, while 41.6% (n = 221) pursued studies in the sciences. As for duration of enrollment at the university, 36.7% of students have been enrolled for less than 1 year, 27.3% have been enrolled for 1–2 years, 19.8% have been enrolled for 3–4 years, and 16.2% have been enrolled for more than 4 years.

**Table 1:** Participants’ demographic profile.

Variables	Level	Frequency	Percentage (%)
Gender	Male	209	39.4
	Female	322	60.6
Age group	18–24	348	65.6
	25–30	159	29.9
	31+	24	4.5
Educational level	Undergraduate	416	78.3
	Postgraduate	115	21.7
Area of study	Liberal arts	310	58.4
	Sciences	221	41.6
Duration of current enrollment	Less than 1 year	195	36.7
	1–2 years	145	27.3
	3–4 years	105	19.8
	More than 4 years	86	16.2

### Research process

Our research process consisted of several stages, starting with the analysis of the quantitative data. We used SPSS 26 and Amos 28 for the initial data analysis to explore the factors influencing students’ overall satisfaction with the internationalization practices at their universities. These software tools enabled us to perform statistical analyses, including correlation and SEM analysis, to identify key variables that influenced student satisfaction. Following the quantitative analysis, we employed topic modeling techniques using R programming to gain deeper insights into the data. Topic modeling is a form of unsupervised machine learning that enables the identification of underlying themes and patterns in large datasets, particularly those derived from open-ended responses. By applying this method, we aimed to uncover insights that may not have been explicitly addressed by the survey questions but were still relevant to the study’s objectives.

## Data analysis and results

### Reliability, validity, and correlation

Table 2 presents the descriptive statistics and factor loadings for each measurable item. All factor loadings exceed 0.7, providing evidence of convergent validity^63^. To assess reliability, Cronbach’s alpha (CA) and construct reliability (CR) are used. As shown in Table 2, the CA and CR values for each construct exceed the recommended threshold of 0.7. Additionally, the average variance extracted (AVE) for all factors is greater than 0.6, further supporting the validity of the constructs. Table 3 shows that the correlations between constructs are all below 0.8, and the square root of the AVE for each construct surpasses the corresponding inter-construct correlations, demonstrating discriminant validity. Furthermore, the Heterotrait-Monotrait (HTMT) ratio is applied to assess discriminant validity by comparing the average correlation between indicators of different constructs (heterotrait) to the average correlation between indicators of the same construct (monotrait). A threshold of < 0.9 indicates that constructs are distinct^64^. In this study, all HTMT values (Table 4) fall below 0.9, indicating no issues with discriminant validity^64^. Taken together, the results of the reliability, convergent validity, and discriminant validity assessments confirm the robustness of the measurement properties and justify the subsequent evaluation of the structural models^65^.

**Table 2:** Factor loading, reliability, and validity.

Construct	Item	Factor loadings	Cronbach’s alpha	AVE	C.R.
Curriculum internationalization	Q1	0.838	0.908	0.712	0.908
	Q2	0.838			
	Q3	0.853			
	Q4	0.846			
Language support	Q1	0.823	0.871	0.631	0.873
	Q2	0.79			
	Q3	0.757			
	Q4	0.807			
Mobility program	Q1	0.854	0.885	0.721	0.886
	Q2	0.865			
	Q3	0.828			
Global reputation of foreign universities	Q1	0.859	0.826	0.618	0.829
	Q2	0.742			
	Q3	0.753			
Campus environment	Q1	0.814	0.916	0.690	0.918
	Q2	0.835			
	Q3	0.83			
	Q4	0.866			
	Q5	0.808			
Student management styles	Q1	0.946	0.975	0.879	0.973
	Q2	0.968			
	Q3	0.951			
	Q4	0.905			
	Q5	0.919			
Perceived equality	Q1	0.854	0.938	0.742	0.935
	Q2	0.869			
	Q3	0.881			
	Q4	0.877			
	Q5	0.826			
Degree of cultural integration	Q1	0.88	0.892	0.735	0.892
	Q2	0.885			
	Q3	0.804			
Student satisfaction	Q1	0.86	0.924	0.754	0.925
	Q2	0.862			
	Q3	0.899			
	Q4	0.853			

**Table 3:** Inter-correlation (square root of AVE on diagonal).

Variable	CI	LS	MB	GRFU	CE	SMS	PE	DCI	SS
Curriculum internationalization	**0.844**	.299^**^	.497^**^	.635^**^	.722^**^	.368^**^	.666^**^	.308^**^	.779^**^
Language support	.299^**^	**0.800**	.277^**^	.274^**^	.365^**^	.221^**^	.346^**^	.658^**^	.331^**^
Mobility program	.497^**^	.277^**^	**0.849**	.397^**^	.487^**^	.062	.465^**^	.319^**^	.531^**^
Global reputation of foreign universities	.635^**^	.274^**^	.397^**^	**0.786**	.632^**^	.343^**^	.529^**^	.261^**^	.665^**^
Campus environment	.722^**^	.365^**^	.487^**^	.632^**^	**0.831**	.387^**^	.693^**^	.321^**^	.739^**^
Student management styles	.368^**^	.221^**^	.062	.343^**^	.387^**^	**0.938**	.432^**^	.183^**^	.364^**^
Perceived equality	.666^**^	.346^**^	.465^**^	.529^**^	.693^**^	.432^**^	**0.861**	.326^**^	.716^**^
Degree of cultural integration	.308^**^	.658^**^	.319^**^	.261^**^	.321^**^	.183^**^	.326^**^	**0.857**	.335^**^
Student satisfaction	.779^**^	.331^**^	.531^**^	.665^**^	.739^**^	.364^**^	.716^**^	.335^**^	**0.868**

**p < .01.

**Table 4:** Heterotrait–Monotrait ratio of correlations.

Variable	CI	LS	MB	GRFU	CE	SMS	PE	DCI	SS
Curriculum internationalization	–								
Language support	0.336	–							
Mobility program	0.554	0.315	–						
Global reputation of foreign universities	0.736	0.322	0.464	–					
Campus environment	0.792	0.408	0.541	0.728	–				
Student management styles	0.391	0.243	0.066	0.384	0.410	–			
Perceived equality	0.722	0.385	0.513	0.605	0.747	0.452	–		
Degree of cultural integration	0.342	0.749	0.360	0.303	0.353	0.197	0.358	–	
Student satisfaction	0.850	0.371	0.588	0.764	0.802	0.384	0.770	0.369	–

### Model fit and hypothesis testing

The structural model fit was evaluated using AMOS 26.0, a software tool designed for testing complex theoretical causal relationships among multiple variables. The statistical results indicate that the data fit the model adequately, as evidenced by the following metrics: χ^2^ = 1601.979, degrees of freedom (df) = 559, χ^2^/df = 2.865, *p*-value < 0.001, CFI = 0.942, NFI = 0.914, GFI = 0.858, AGFI = 0.831, RMSEA = 0.059, and SRMR = 0.0339. These indices collectively suggest a favorable fit of the proposed model to the observed data, supporting the validity of the theoretical framework under investigation.

The results of the hypothesis testing, as presented in Table 5, indicate that among the common practice variables, only the variable of language support was found to be non-significant (β = − 0.026, *p* = .521), thus leading to the conclusion that H2 is not supported. In contrast, the remaining common practice variables demonstrated varying levels of significance. Curriculum internationalization emerged as the most influential determinant of students’ overall satisfaction with internationalization practices (β = 0.367, *p* < .001), thus supporting H1. Following this, the global reputation of the foreign universities was identified as the second most significant factor (β = 0.189, *p* < .001), providing robust support for H4. Additionally, both campus environment (β = 0.149, *p* = .007) and mobility programs (β = 0.107, *p* = .003) were found to significantly affect student satisfaction, lending support to H3 and H5, respectively. As for the contextual factors, only perceived equality was shown to be a significant determinant of overall satisfaction (β = 0.212, *p* < .001), thereby supporting H7. On the other hand, student management styles (β = 0.020, *p* = .936) and degree of cultural integration (β = 0.043, *p* = .336) were not found to significantly influence student satisfaction, meaning that H6 and H8 were not supported.

**Table 5:** Standardized structural estimates.

Paths			Hypothesis	Standardized estimate	C.R.	*P*	Results
Student satisfaction	←	Global reputation of foreign universities	H1	0.189	3.902	0.000***	Support
Student satisfaction	←	Campus environment	H2	0.149	2.697	0.007**	Support
Student satisfaction	←	Perceived equality	H3	0.212	4.583	0.000***	Support
Student satisfaction	←	Curriculum internationalization	H4	0.367	6.385	0.000***	Support
Student satisfaction	←	Student management styles	H5	0.020	0.081	0.936	Not support
Student satisfaction	←	Language support	H6	− 0.029	− 0.641	0.521	Not support
Student satisfaction	←	Mobility program	H7	0.107	3.006	0.003**	Support
Student satisfaction	←	Degree of cultural integration	H8	0.043	0.962	0.336	Not support

***p < .001, **p < .01, *p < .05.

### Topic modeling

To process the open-response text data, which consists of 531 reviews, the text was initially translated using ChatGPT. This AI-based translation was chosen for its advanced ability to capture the contextual meanings of the reviews. After translation, each output was manually reviewed and verified to ensure the accuracy and reliability of the translated content. The text data was then pre-processed in several stages to prepare it for analysis. First, the suggestions related to internationalization practices were converted into a text corpus, and all text was converted to lowercase to maintain uniformity. Numbers and punctuation were removed to eliminate irrelevant elements, and lemmatization was applied to reduce words to their root forms, standardizing the vocabulary. Next, common stopwords were removed using a specialized stopword list tailored to the context of the reviews, and excessive whitespace was stripped to further clean the data. Residual noise, such as non-alphanumeric characters, was removed through a regular expression, ensuring the text was free from unwanted symbols. After preprocessing, 501 reviews remained for the subsequent topic modeling analysis.

**Fig. 2:**
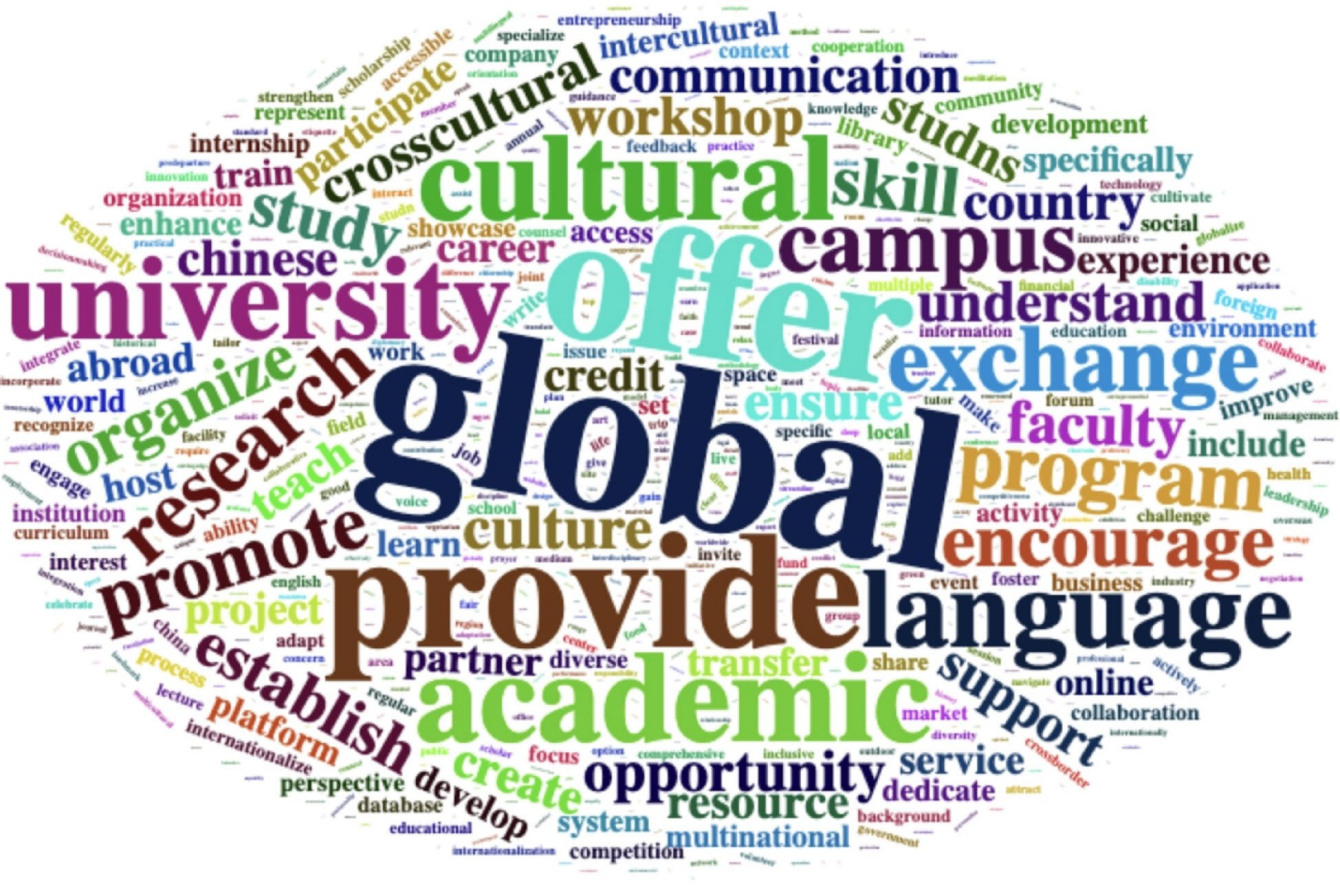
Word cloud of students’ responses. This figure is created using the “wordcloud” package in R programming version 4.4.2. https://cran.r-project.org/web/packages/wordcloud/wordcloud.pdf.

Figure 2 provides an overview of the text data in the form of a word cloud, visually highlighting the most frequently occurring terms. Prominent keywords such as ‘global,’ ‘cultural,’ ‘academic,’ ‘exchange,’ and ‘language’ stand out, reflecting the key themes that emerge from the students’ responses. These terms suggest a strong emphasis on concepts related to international collaboration, cultural diversity, and academic exchange, indicating that students perceive these elements as central to their experiences and aspirations.

**Fig. 3:**
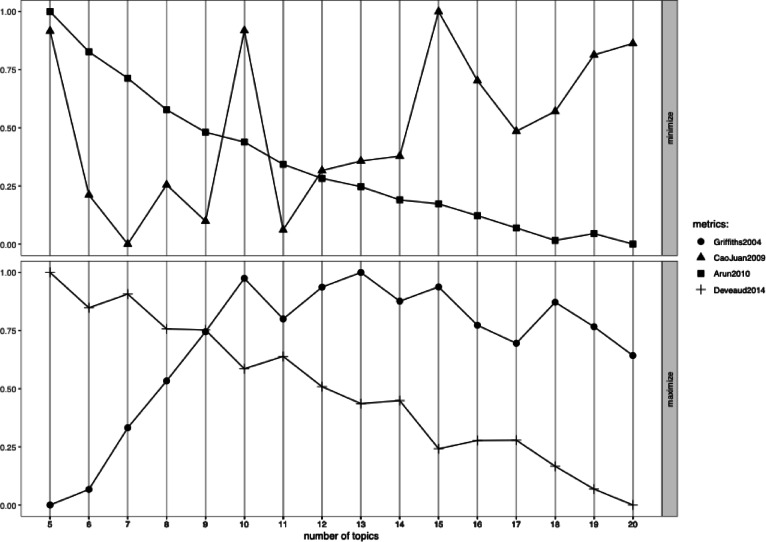
Evaluation metrics.

After preprocessing the data, we employed the “ldatuning” and “topicmodels” packages to identify the optimal number of topics for analysis. To evaluate the best model, we applied four different criteria. As shown in Fig. 3, the CaoJuan2009 and Griffiths2004 criteria were particularly sensitive to variations in the number of topics. Based on the tuning results, both the 10-topic and 15-topic models demonstrated superior performance. Following this, we conducted a qualitative evaluation of the model outputs, focusing on interpretability and the coherence of concepts within each topic. The 10-topic model lacked sufficient granularity, as we observed several concepts grouped within a single topic. As a result, we chose to proceed with the 15-topic model, which provided clearer and more distinct conceptual themes for further analysis.

**Fig. 4:**
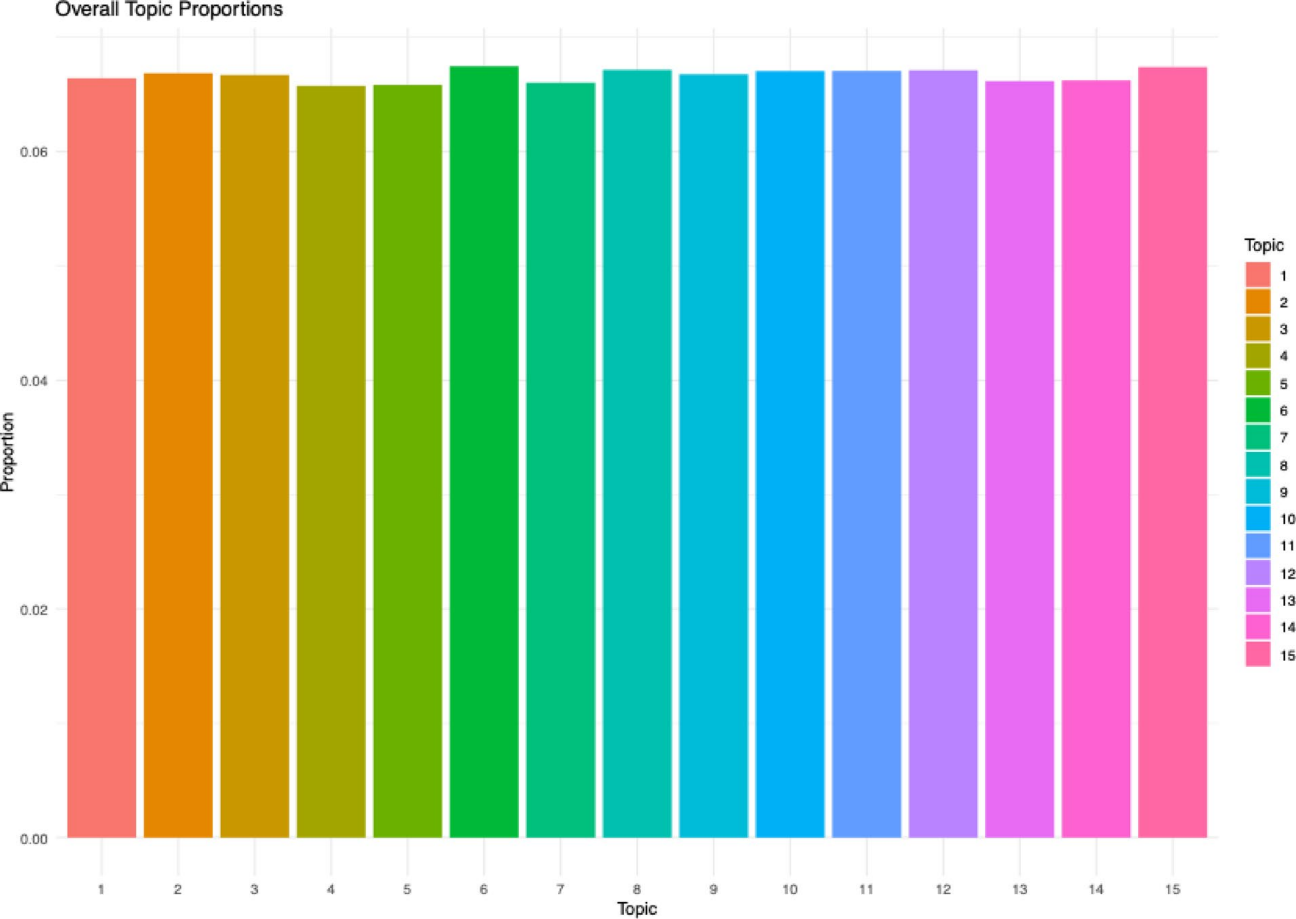
Average topic proportion.

The results of the topic modeling analysis are presented in Table 6. To assign meaningful labels to each topic, the top *n* words ranked by their phi-values were utilized. These words were interpreted to identify the underlying concepts they collectively represented. All authors participated in the labeling process, which involved a multi-step approach. Initially, the top 20 words for each topic were analyzed to propose preliminary labels. Subsequently, the most representative text excerpts associated with each topic were examined to contextualize the words and validate the initial labels. The final topic labels were confirmed only after reaching a consensus among all authors. In addition to the internal validation process, the findings were compared with existing literature on internationalization in higher education. Notably, three topics identified in this study were found to be previously unreported in the literature, as indicated by the mark “N” in Table 5. Figure 4 illustrates the average proportion of each topic across the dataset. While the differences in topic proportions are relatively minor, Topics 6 and 15 exhibit slightly higher prevalence compared to the others. This suggests that these topics may represent more prominent themes within the dataset.

**Table 6:** Topic modeling results.

Topic No.	Top words	Topic label	Literature
1	Cultural, culture, organize, country, experience, social, event, share, student, diversity	In-campus cultural activities	Y
2	Student, ensure, create, resource, chinese, background, internship, curriculum, integrate, study	Academic resources	Y
3	Offer, skill, workshop, communication, crosscultural, specific, focus, write, business, voice	Intercultural communication skills	Y
4	Credit, transfer, language, system, process, set, information, experience, accessible, multiple	Academic credit transfer	Y
5	Student, international, train, comprehensive, intercultural, invite, orientation, outdoor, organize, learn	International student exchange preparation	N
6	Student, opportunity, chinese, specifically, career, service, support, organization, enhance, project	Career support for international students in local markets	Y
7	Research, faculty, understand, teach, promote, engage, abroad, foster, make, specialize	International research development	Y
8	Academic, international, improve, community, context, project, culture, participate, establish, world	Global academic communities	Y
9	Student, establish, online, participate, strengthen, field, live, world, student, art	Participation in international virtual events	N
10	Campus, university, develop, environment, diverse, crosscultural, intercultural, feedback, abroad, space	Campus infrastructure	Y
11	International, encourage, interest, local, student, innovation, showcase, platform, adapt, work	Opportunities for global exposure	N
12	Global, perspective, platform, encourage, activity, market, annual, lecture, offer, job	Global job opportunities	Y
13	Provide, students, host, offer, actively, student, studn, entrepreneurship, dedicate, issue	Global entrepreneurship support	N
14	University, study, multinational, student, represent, ability, partner, learn, include, global	Student representation	Y
15	Exchange, program, language, promote, institution, regularly, train, support, tutor, life	Student exchange programs	Y

### Topic interpretation

**Fig. 5:**
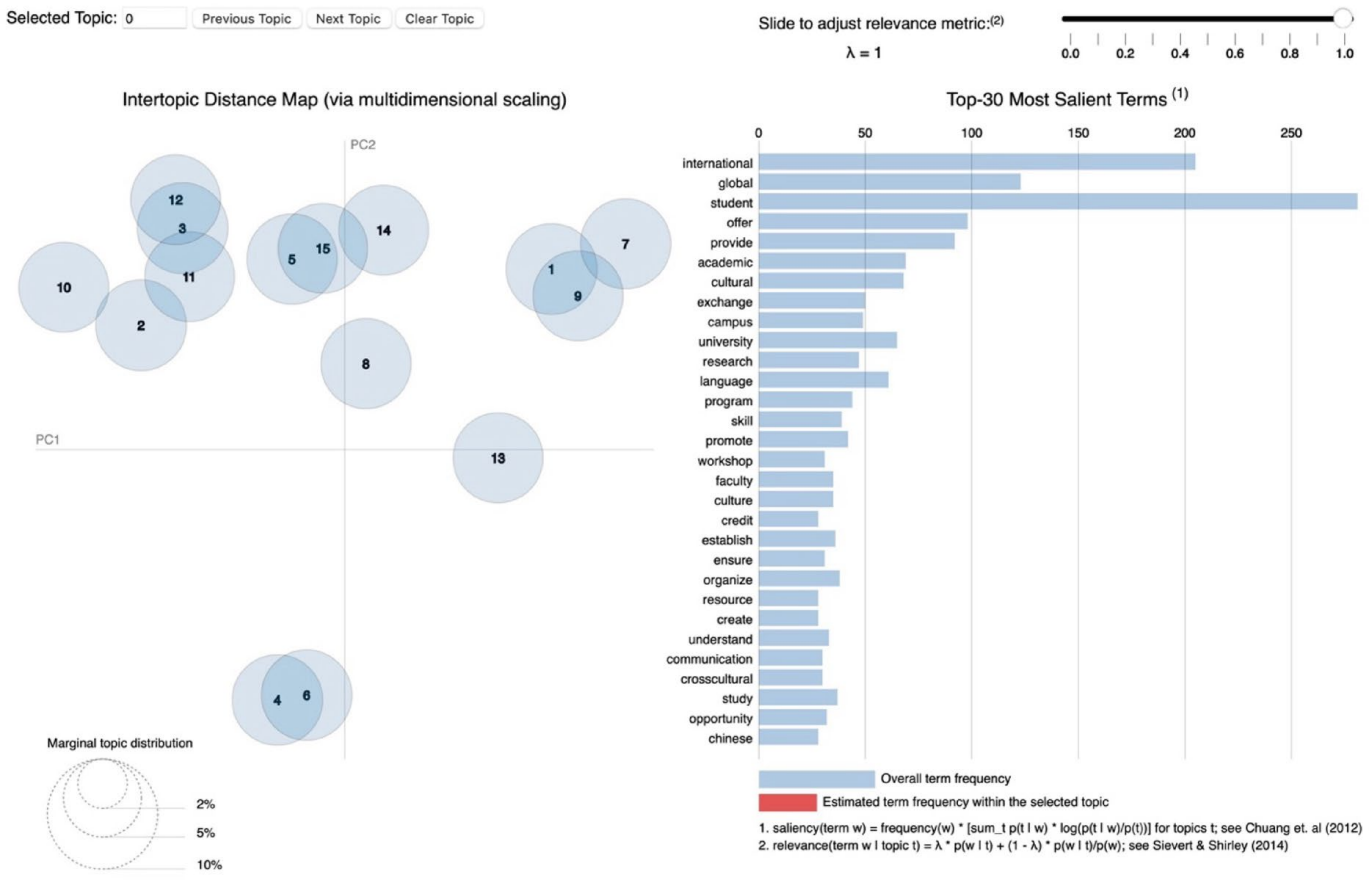
Topic distribution.

Based on Fig. 5, we interpret the topics by analyzing the clusters represented in the fig. Topics that show overlapping areas indicate that their content is frequently discussed together, suggesting a close relationship or thematic connection between them.

Topics 8 and 13 are the only ones that do not overlap with any other topics. Topic 8 focuses on the enhancement and support of academic communities with an international perspective. Its emphasis lies in creating environments where diverse cultural perspectives are integrated, fostering improved academic participation and collaboration on a global scale. Topic 13, on the other hand, highlights the importance of SFCUs actively providing and hosting programs, resources, and events specifically dedicated to supporting global entrepreneurship. The representative text suggests that these initiatives should offer practical opportunities and ensure students are adequately prepared to engage in entrepreneurial activities on an international stage.

Topics 4 and 6 share a common focus on student empowerment and skill development. Topic 4 highlights students’ suggestions for improving the academic credit transfer system in SFCUs. The proposed system should accommodate a range of scenarios, including transfers between different institutions and countries, while addressing potential challenges such as language barriers. In addition, Topic 4 points out the need for enhanced career support for international students in the local (Chinese) job market.

Topics 1, 7, and 9 form a cluster centered around cultural exchange, each contributing to the broader goals of internationalization and global engagement within universities. Topic 1, titled ‘In-campus Cultural Activities,’ focuses on organizing and promoting events that celebrate cultural diversity and encourage social interaction among students. These activities offer students the opportunity to share their cultural backgrounds, experience other cultures, and build relationships within the university. Topic 7, ‘International Research Development,’ highlights the importance of advancing research through global collaboration. This topic emphasizes students’ desire for cross-border partnerships to drive innovation, share knowledge, and address global challenges through collaborative research initiatives. Topic 9, ‘Participation in International Virtual Events,’ indicates the value of enabling students to participate in online events that connect them to the global community. These virtual events, often conducted in real time, allow students to engage with diverse perspectives and ideas from around the world, helping them strengthen their skills and deepen their knowledge in their respective fields, such as art, science, and technology.

Topics 5, 14, and 15 are interconnected, with Topic 15 closely linked to both Topic 5 and Topic 14. Topic 5 emphasizes the need for well-organized training to prepare students for their international exchange programs. This includes intercultural training, orientation sessions, and opportunities to learn from experts and peers. The goal is to foster personal and academic growth, enabling students to make the most of their exchange experience. Topic 15 reflects students’ interest in improving and expanding opportunities related to student exchange programs. It goes beyond simply having exchange programs and delves into the quality of the experience. Specifically, the top words suggest that students are not simply advocating for more exchange programs but rather for high-quality programs with sufficient support systems, particularly in language acquisition and cultural integration. Topic 14 advocates for the inclusive and equitable participation of all students in university governance and decision-making processes. Topic 14 underscores the significance of ensuring adequate representation of multinational students, which fosters a sense of belonging and empowerment.

Topics 3, 11, and 12 are closely interconnected in their focus on strengthening students’ global engagement. Topic 3 centers on the essential skills required for success in a global environment, with students particularly emphasizing the importance of intercultural communication, which is difficult to acquire without firsthand international experience. Building on this, Topic 11 advocates for universities to create institutional platforms that support international exposure and innovation. It underscores the value of integrating global perspectives with locally relevant experiences to help students navigate diverse cultural settings and prepare for international careers. Similarly, Topic 12 highlights students’ expectations for universities to actively facilitate access to global job markets by organizing events such as annual career fairs and employer lectures featuring international companies.

Topics 2 and 10 are slightly connected. Topic 2 suggests the development and provision of comprehensive support systems to enhance students’ learning experiences. It emphasizes the need to create and integrate resources that cater to the diverse backgrounds and needs of students. Topic 10 advocates for the development of physical and organizational structures that create a globally engaged environment for students and faculty. This includes designing spaces that facilitate cross-cultural interactions, gathering feedback to ensure facilities meet the needs of a diverse community, and integrating various cultural elements into campus planning.

## Discussion and implications

### Discussion of major findings

This study investigated the determinants of students’ overall satisfaction with internationalization practices in SFCUs, focusing on common institutional practices and contextual factors. The findings offer vital information about the relative importance of these variables, with implications for theory, institutional strategy, and future research.

The results indicate that curriculum internationalization (H1) is the strongest predictor of students’ satisfaction. This aligns with theories that emphasize the central role of academic experiences in internationalization outcomes^66,67^. In the context of SFCUs, a globally oriented curriculum directly enhances students’ academic engagement and their perception of the institution’s commitment to global education. This finding highlights the importance of educational quality and relevance in improving satisfaction, even more so than infrastructure or extracurricular factors. Global reputation (H4) emerged as the second most influential factor, consistent with studies linking institutional prestige to student decision-making and satisfaction^68^. For students in SFCUs, reputation likely serves as a proxy for perceived quality and employability outcomes^69^, validating their investment in international education. This is particularly relevant in the Chinese context, where institutional reputation significantly influences student choices and expectations^70^.

The weaker but significant effects of the campus environment (H3) and mobility programs (H5) further highlight the complexity of student satisfaction. The campus environment reflects the importance of providing a welcoming and functional setting for international and domestic students alike^71^. Consistent with Guo and Chase’s^72^ findings, the campus environment is regarded as a cornerstone of internationalization in higher education. This is because a campus environment that promotes cross-cultural communication is conducive to students’ acquisition of necessary skills, enabling them to move forward smoothly and achieve success in a globalized environment^73^. Mobility programs, meanwhile, align with experiential learning theories, which suggest that direct exposure to global contexts enhances satisfaction through skill development and cultural immersion^74^. These programs are particularly valuable in Sino-foreign partnerships, as they provide students with opportunities to experience the educational systems and cultures of partner institutions abroad, which is essential for developing global citizenship and sociolinguistic awareness^75^.

Among the contextual factors examined, the perceived degree of equality among people turned out to be the sole significant factor influencing students’ satisfaction (H7). This finding aligns with social justice frameworks in international education, which emphasize equity as a core dimension of inclusive and sustainable institutional practices^31,59^. Herr et al.^76^ suggest that students’ perceptions of fair treatment extend not only to formal policies but also to everyday interactions and access to institutional resources. This is particularly evident in the university environment of China, as concerns over international students receiving special treatment have led to dissatisfaction among local students^77^. Therefore, fair treatment appears to heavily influence student satisfaction. Conversely, language support (H2) was not a significant predictor, contrasting with literature emphasizing language barriers as a critical challenge^78^. In SFUCs, this non-significance may be explained by two key factors related to the sample and context. First, the student population likely possessed high pre-existing English proficiency, reducing their perceived need for support. More critically, our topic modeling analysis revealed a student preference for immersive English environments, with fewer mentions of formal teaching settings. This suggests the non-significance of H2 could also stem from a misalignment between the design of formal class and students’ preferred, immersion-based learning strategies. As Ozverir et al.^79^ suggest, structured language classes often fail to replicate the complexity and spontaneity of real-life conversations, leading to a gap between theoretical knowledge and practical application.

Student management styles (H6) and cultural integration (H8) did not emerge as statistically significant predictors of students’ overall satisfaction. One possible explanation is that internationalized management practices, often rooted in Western educational ideologies, may not resonate with the cultural values and learning expectations of Chinese students^80^. Although SFCUs have adopted global models such as student-centered governance or flexible credit systems to align with international standards, these reforms may lack substantive alignment with local educational traditions^81^. For example, initiatives like cross-cultural extracurricular programs or decentralized student support structures may be perceived more as symbolic compliance with global norms than as meaningful changes, particularly when they do not address students’ instrumental goals or conflict with entrenched Confucian ideals of hierarchy and meritocracy^59^. Ball et al.^82^ describe this mismatch as the “enactment gap,” where local implementation reinterprets globally inspired policies. In such cases, institutional actors, including administrators and faculty, may modify these policies to fit prevailing norms and operational constraints. This can significantly weaken the intended impact of internationalized governance, particularly when local stakeholders fail to fully prioritize the underlying goals.

The non-significance of cultural integration (H8) is particularly noteworthy, especially given that these are local Chinese students. One might expect a greater emphasis on maintaining Chinese culture within these Sino-foreign ventures. While Guo et al.^23^ argue that internationalization often marginalizes local cultural agency, our findings suggest a paradox of cultural pragmatism among Chinese students. Topic modeling reveals that students prioritize cross-cultural instrumentalism (e.g., the topic ‘Intercultural communication skills’) over symbolic cultural preservation. This aligns with Bourdieu’s concept of cultural capital, where students may view cultural exchange as a means to enhance global competitiveness rather than an end in itself^83^. This pattern can also be understood through the lens of mimetic isomorphism, a concept from DiMaggio and Powell’s^84^ Institutional Isomorphism theory. To gain legitimacy, SFCUs often follow the practices of well-established Western institutions^25^. This leads them to align their programs with international norms and global expectations, frequently placing greater emphasis on globally oriented activities while giving less attention to local cultural content^27^. Consequently, institutional strategies tend to focus on global engagement instead of cultural preservation. This orientation aligns with student preferences as well, as many are more drawn to the international and cross-cultural dimensions of their education than to the integration of local cultural elements^85^.

### Theoretical implications

This study offers several key theoretical contributions to the literature on higher education internationalization. This research empirically validates crucial determinants of student satisfaction within the specific context of SFCUs, which is an invaluable addition to the predominantly qualitative approaches of previous research (e.g^86–88^. , . A key contribution lies in the incorporation of contextual variables, revealing context-specific findings that diverge from some established theoretical frameworks. Specifically, this research challenges the widely accepted argument that integrating local culture into the curriculum is a crucial aspect of internationalization, particularly in non-Western educational contexts. While many scholars argue that incorporating local cultural values into the curriculum supports internationalization goals^9,71,89^, this study did not provide strong evidence to support this claim in the context of SFCUs. This finding suggests a reconsideration of the assumption that local cultural integration is essential for internationalization strategies. Such integration might need to be adapted to the specific educational context and aligned with the needs and preferences of the local student population.

The study’s integration of topic modeling, complementing the structured survey data, provides valuable methodological insights. This approach allows for the exploration of diverse student perceptions that traditional survey instruments may overlook^90^. Specifically, topic modeling can uncover latent patterns within textual data, offering a richer understanding of student experiences. This methodological contribution has the potential to inform the development of more comprehensive and sensitive scales for future research on student perceptions of internationalization. By leveraging these insights, future studies can refine existing theoretical frameworks.

### Practical implications

The findings highlight several strategic priorities for SFCUs that aim to enhance student satisfaction with their internationalization efforts. First, curriculum internationalization should be a central focus, with universities aligning their programs to global standards while preserving academic rigor. This emerged as the strongest predictor of satisfaction, suggesting the need for closer collaboration with international partner institutions to co-develop courses, integrate global case studies, and promote interdisciplinary learning that equips students for transnational careers.

Second, leveraging institutional reputation is vital, especially in the Chinese context, where prestige plays a significant role in influencing perceptions of quality and employability. By strategically enhancing their brand through partnerships with globally ranked universities and showcasing successful alumni, SFCUs can strengthen their position as a competitor in the international higher education sector.

Third, the importance of perceiving equality urgently needs to be translated into more specific policy actions. From the theoretical framework of social justice, SFCUs need to develop policies to ensure that both domestic and international students can have equal access to academic resources, financial support, and extracurricular activity opportunities. For instance, establishing unified scholarship evaluation criteria, standardizing inclusive access channels for support services, and ensuring fair representation of students from diverse backgrounds in student organization management. In addition, it is suggested to conduct regular inspections on educational equity and establish direct feedback mechanisms for students’ opinions to effectively monitor and address issues of actual treatment differences or unequal opportunities.

Fourth, although language support was not a significant predictor of satisfaction, this may be attributed to the relatively high proficiency of students in these environments. Nonetheless, SFCUs still should provide adequate language support services while emphasizing academic and cultural skills to help students prepare for globalized careers. While campus environment and mobility programs were secondary predictors, they are important to overall student satisfaction, including internationalized facilities and expanding opportunities for study-abroad exchanges.

Lastly, SFCUs should carefully evaluate the approach to cultural preservation. It is suggested that institutions should focus on fostering cross-cultural competence through different measures rather than emphasizing cultural integration as a mandatory component. This shift aligns with students’ preference for practical, global engagement rather than symbolic cultural integration. Cultural integration could be offered as an elective or as part of selected courses, allowing students to choose their level of involvement based on personal interest, rather than requiring it as a mandatory aspect of their education.

## Conclusion

In conclusion, our research sheds light on what truly matters to students experiencing internationalization efforts within SFCUs. The results confirmed that when students consider internationalization, a globally minded curriculum and a strong international reputation matter the most, followed by the importance of an internationalized campus and exchange opportunities. Perceived equality emerged as a critical contextual factor, whereas language support exhibited a non-significant relationship with satisfaction. In contrast to some established assumptions, the research also revealed a divergence from the perceived importance of integrating local culture into the curriculum within this specific context. The methodological integration of topic modeling further enriches the analysis, offering insights beyond those typically obtained through traditional survey methodologies. From a practical perspective, this study suggests that, to enhance student satisfaction, SFCUs should prioritize practical measures that align closely with students’ expectations. Rather than adopting a universal approach based on Western models, institutions should tailor their internationalization strategies to the specific needs and contexts of their student population.

### Limitations and suggestions for future research

This study has several limitations that should be acknowledged, which provide opportunities for future research in the field of internationalization in higher education. First, the research exclusively focuses on the perspectives of domestic Chinese students at SFCUs, due to the distinct preferences and experiences of domestic versus international student populations. This narrow focus may limit the generalizability of the findings, particularly when considering the perspectives of international students or other stakeholders, such as faculty or administrative staff. Future research could expand the scope to include these groups to gain a more comprehensive understanding of the factors influencing satisfaction with internationalization practices. Second, this study only examines universities located in economically developed regions of China. These regions may differ significantly from less developed areas in terms of infrastructure, resources, and student demographics. Future studies could compare universities in both developed and less developed regions of China to explore whether regional differences affect students’ perceptions of internationalization. Additionally, comparative research involving SFCUs in other countries could provide valuable insights into the cross-cultural applicability of the identified factors. This would help to determine if the determinants of student satisfaction in the context of internationalization are consistent across different national context.

## Data Availability

The data supporting the findings of this study are available upon reasonable request from the corresponding author.
